# Are Measles More Dangerous in Grown-up Persons Than in Children?

**Published:** 1880-11

**Authors:** Herman Mynter


### Translations.

### ARE MEASLES MORE DANGEROUS IN GROWN-UP PERSONS THAN IN CHILDREN?

FROM THE DANISH BY HERMAN MYNTER, M. D.

It is generally believed that measles are more fatal in grown-up people. Dr. Trier shows, that this is not always the case. During the epidemics on the Faroe islands in 1846 and 1875, the mortality among adults was greater than among children, while in Copenhagen the opposite has been the case for many years. Dr. Trier proves this by counting the number of cases and the number of deaths for the last four epidemics. Between

May 28th and November 2d, 1879, 9,361 cases occurred, of which 319 were adults. The number of deaths for the same time was 212, of which none were adults.—*Nordisk Medicinsk Archiv.*

